# Automatic Classification of Tremor Severity in Parkinson’s Disease Using a Wearable Device

**DOI:** 10.3390/s17092067

**Published:** 2017-09-09

**Authors:** Hyoseon Jeon, Woongwoo Lee, Hyeyoung Park, Hong Ji Lee, Sang Kyong Kim, Han Byul Kim, Beomseok Jeon, Kwang Suk Park

**Affiliations:** 1 The Interdisciplinary Program for Bioengineering, Seoul National University, Seoul 03080, Korea; nulpurunhs@bmsil.snu.ac.kr (H.J.); hongjidan@bmsil.snu.ac.kr (H.J.L.); skkim@bmsil.snu.ac.kr (S.K.K.); hahanbyul@bmsil.snu.ac.kr (H.B.K.); 2 Department of Neurology and Movement Disorder Center, Seoul National University Hospital, Seoul 03080, Korea; w2pooh@daum.net (W.L.); 0907bluelove@naver.com (H.P.); brain@snu.ac.kr (B.J.); 3 Department of Biomedical Engineering, Seoul National University College of Medicine, Seoul 03080, Korea

**Keywords:** tremor, UPDRS, automatic scoring, Parkinson’s disease, wearable device, machine learning algorithm

## Abstract

Although there is clinical demand for new technology that can accurately measure Parkinsonian tremors, automatic scoring of Parkinsonian tremors using machine-learning approaches has not yet been employed. This study aims to fill this gap by proposing machine-learning algorithms as a way to predict the Unified Parkinson’s Disease Rating Scale (UPDRS), which are similar to how neurologists rate scores in actual clinical practice. In this study, the tremor signals of 85 patients with Parkinson’s disease (PD) were measured using a wrist-watch-type wearable device consisting of an accelerometer and a gyroscope. The displacement and angle signals were calculated from the measured acceleration and angular velocity, and the acceleration, angular velocity, displacement, and angle signals were used for analysis. Nineteen features were extracted from each signal, and the pairwise correlation strategy was used to reduce the number of feature dimensions. With the selected features, a decision tree (DT), support vector machine (SVM), discriminant analysis (DA), random forest (RF), and *k*-nearest-neighbor (*k*NN) algorithm were explored for automatic scoring of the Parkinsonian tremor severity. The performance of the employed classifiers was analyzed using accuracy, recall, and precision, and compared to other findings in similar studies. Finally, the limitations and plans for further study are discussed.

## 1. Introduction

Despite the growing body of literature about tremor quantification for objective and quantitative diagnosis, automatic scoring of the Parkinsonian tremor severity using machine-learning algorithms for routine clinical assessments has yet to be investigated. Seeking to address this deficit in research, this paper expounds upon a machine-learning approach to objectively measure and accurately evaluate Parkinsonian tremors using the Unified Parkinson’s Disease Rating Scale (UPDRS). The aim of this study is to connect the automatic scoring system and current clinical rating scale in a more natural manner in order to attain a more objective and sophisticated diagnosis and to provide clinical convenience.

### 1.1. Background

As PD is a movement disorder; its symptoms mainly show clinical abnormalities of movements including tremor bradykinesia, rigidity, and postural instability [1]. A Parkinsonian tremor is defined as an involuntary, rhythmic, oscillatory, and back-and-forth action [2]. It is certainly one of the most disabling symptoms of PD. In particular, the ability to perform functional upper-limb motor tasks is crucial for most activities in daily life, and consequently, patients with a Parkinsonian tremor experience difficulties on a daily basis, either physically or psychologically. Therefore, the evaluation of a tremor is crucial when seeking to diagnose, treat, and manage this disease.

The assessment of a Parkinsonian tremor is usually accomplished by using rating scales. Various clinical rating scales have been used to quantify the symptoms of PD patients, of which the UPDRS is one of the best-known and most widely used methods [3]. The UPDRS provides comprehensive information about PD patients’ disabilities for understanding the state of each patient. On the basis of the UPDRS, neurologists use digits to evaluate the severity of a total of 42 motor abilities by observing patients’ conditions and their performance at various tasks [4]. The digits for assessment range from 0 to 4. Normal conditions without any symptoms are rated as 0, and the most severe conditions are awarded a score of 4. An example is provided in Table 1 for the resting tremor examination.

Although this method is widespread, the results obtained by this approach depend on the observer’s opinion. Ratings by trained observers and the patients’ own ratings have been surprisingly low, but there may also be poor reliability between raters [5,6]. In addition, this approach is not suitable to follow up on patients’ conditions in their daily lives since the clinical rating scales are employed only during routine clinical visits, despite PD being notorious for its fluctuations and its dependence on patients’ conditions [7]. Therefore, clinical demand for the advent of new wearable technology that can objectively collect and quantitatively analyze data has arisen, and, by extension, has also arisen for long-term evaluations and the follow-up of PD abnormalities [7].

### 1.2. Related Research

Many previous studies have proposed measurable technologies and the analysis of the characteristics of Parkinsonian tremors using various techniques [8,9,10,11,12,13,14,15,16,17,18,19]. Research groups have mainly used movement sensors such as accelerometers or gyroscopes [10,11,12,13,14,15,16,17,18,19,20] and electromyography (EMG) [8,9,13,16] for quantifying tremors, long-term monitoring, detecting tremors, and differentiating between Parkinsonian tremors and other diseases such as essential tremors or those of healthy people. In research studies using EMG, Parkinsonian hand tremors were demarcated with a high sensitivity from healthy controls by extracting features from EMG signals or from both EMG and acceleration [8,9,16]. The effects of aging on the regularity of physiological tremors were also determined by calculating the regularity, coherence, and modal frequency [13]. Several other studies have proposed objective methods to quantify tremors using an accelerometer, a gyroscope, and a smartphone. Features from the signals of these sensors showed high and significant correlations with the UPDRS [10,11,18]. In a study by Meigal et al. [9], the linear and nonlinear tremor characteristics of PD from acceleration were analyzed, and Kostikis et al. [15] quantified PD-induced hand tremors. In their study, PD patients and healthy volunteers were identified with high sensitivity and specificity by comparing six different machine-learning approaches [15]. To differentiate Parkinsonian tremors from essential tremors, a fluctuation ratio was defined from the angular velocity as a proportion of the temporal fluctuations in the tremor [14]. The postural re-emergent tremors of PD were also distinguished from essential tremors using a smartwatch [17]. In [19], the tremors of PD patients treated with deep brain stimulation (DBS) and nontremors were separated using a gyroscope. For long-term monitoring throughout the day, a home-based PD assessment system (KinesiaTM, Cleveland Medical Devices Inc., Cleveland, OH, USA) was proposed for quantitative severity scores that are highly correlated to clinical rating scales for tremors and bradykinesia [21].

Despite these studies obtaining good results for objective and quantitative approaches, most clinicians continue to use the clinical rating scale as a gold standard, and the currently available techniques have not yet found their way into routine clinical assessment [7,22]. Martinez-Manzanera et al. pointed out the gap between current subjective clinical rating scales, which neurologists continue to consider to be more familiar to use, and the scientific and objective assessments of PD symptoms in clinical practice [22]. Therefore, other clinical and alternate strategies are needed to naturally substitute the present clinical rating scales for scientific and objective tools. The basic idea is to develop an automatic scoring system for PD with the ability to obtain an evaluation of the motor status of a patient as close as possible to the neurologists’ evaluations, based on the pervasive UPDRS scoring methods.

Several approaches for automatically evaluating relevant features representative of the UPDRS using motion sensors have been presented for gait and gait-related tasks [23,24,25,26], bradykinesia [22,27], a finger-tapping task [28,29], and tremors [30,31,32]. Among them, studies for the gait and bradykinesia used machine-learning methods, a support vector machine (SVM), and a regression model, respectively, to automatically obtain a score [22,23,24,25,26,27]. Logistic and linear regression models were employed to estimate the UPDRS for the finger-tapping task and tremors [28,29,30,31,32]. These studies reported acceptable performance.

To the best of our knowledge, however, machine-learning approaches have not yet been studied for the automatic scoring of Parkinsonian tremors. In this project, we aim to automatically assign the UPDRS of tremors using several machine-learning classifiers to obtain scientific and objective evaluations of Parkinsonian tremors. We designed, developed, and validated an automatic scoring system for PD that can easily be used in a clinical or home setting. We first describe patient participation, experiments, feature definition, and extraction, as well as multiclassification methods (in Section 2), in this order. Then, the relationships between the extracted features and UPDRS and the performance of the developed systems are explained in Section 3. Section 4 and Section 5 discuss the development considerations, potential limitations, and future directions.

## 2. Methods

### 2.1. Subjects

Eighty-five patients with PD (average age: 65.96 ± 9.19 y, 44 females, 41 males) participated in this study. The patients met the diagnostic guidelines of the United Kingdom Parkinson’s Disease Society Brain Bank criteria. All of these patients visited the Department of Neurology at the Seoul National University Hospital (SNUH) in the Republic of Korea and were diagnosed as experiencing hand tremor symptoms in their daily lives (this does not mean that they definitely exhibited hand tremors during their visit). Patients with leg tremors or dyskinesia were not recruited. This study was conducted according to the principles of the Declaration of Helsinki (2008) with prior approval of the Ethics committee of the Seoul National University Hospital (SNUH).

### 2.2. Wearable Device and Acquisition of Tremor Signals

**Figure 1:**
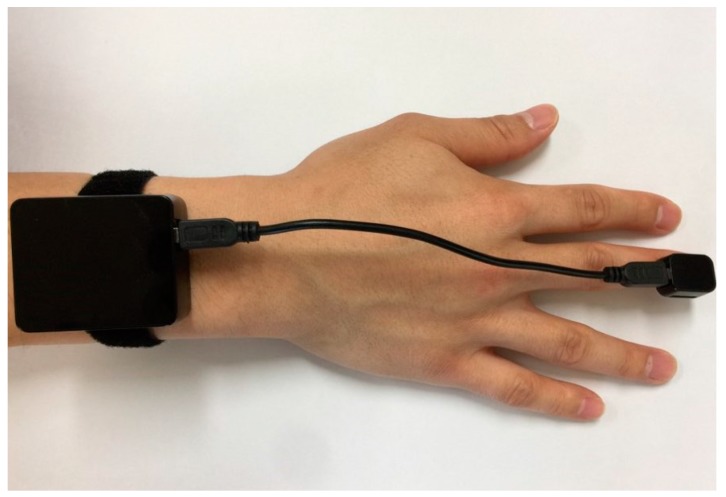
Wrist-watch-type wearable device for measuring tremors.

A wristwatch-type wearable device was designed for this project to measure the acceleration and angular velocity of hand tremors at the same time. This is a small and low-power wireless wearable device, equipped with a triaxis accelerometer sensor (LIS3DSH, STMicroelectronics, Switzerland) and triaxis gyroscope sensor (L3GD20, STMicroelectronics, Switzerland), as shown in Figure 1 and described in Table 2. The accelerometer can measure up to ±16 g along the *X*, *Y*, and *Z* axes, and the gyroscope has ±2000 dps (degrees per second). The operating time of the device is 12 h when the battery is fully charged. The battery is Li-polymer with a capacity of 430 mAh and can be charged via a USB cable.

**Figure 2:**
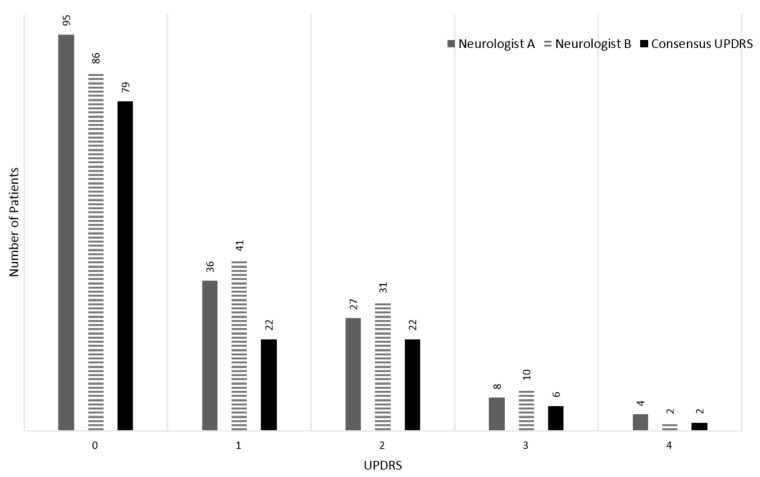
Distribution of the UPDRS scores by two neurologists and the consensus score.

The device was attached to the wrist and the fingertips of the middle fingers of both the right and left hands. We used the signals of the fingers because they are mainly affected by tremor symptoms in most of the PD patients recruited in this study. The sensor position was changed when the patient had more severe tremor symptoms, specifically on another finger. Resting tremor signals were acquired when each patient was comfortably seated in a chair and after their forearms had rested on the arms of the chair for 1 min. A video camera (Panasonic HDC-TM700, 1920 × 1080 HD, 60 frames per second) simultaneously recorded the patients’ hands extended to the front of the patient for later evaluation by neurologists. Both hands were recorded as large as possible within the screen of the camera for enhanced evaluation. Two neurologists, who had at least two years of subspecialty training in movement disorders, independently scored the hand-tremor severity by watching the video on the basis of the guidelines of the UPDRS. In total, 170 recordings of 85 patients’ hands were scored through video. We chose tremor recordings for which the two clinicians were in consensus about each other’s scores to design the automatic scoring system of tremor severity. As a result, 131 tremor recordings (59 and 72 recordings from the right and left hands, respectively) were selected from a total of 170 tremor recordings, and each of them belonged to one of five classes ranging from 0 to 4, with a difference of 1. Figure 2 shows the distributions of the UPDRS scores from two neurologists and the consensus UPDRS score.

### 2.3. Data Analysis

#### 2.3.1. Signal Processing

All signals were recorded at a sampling rate of 125 Hz and streamed via Bluetooth to a computer. After being scaled, the signals were band-pass-filtered between 1 and 16 Hz to remove artifacts such as drift and noise from the main electrical power line using a fifth-order Butterworth filter. Following that, the displacements and angles were calculated by integrating the accelerations twice and integrating the angular velocities once. The acceleration, angular velocity, displacement, and angle signals were segmented before calculating the features. A 30-s signal was segmented from the middle part of the entire signal after removing the first 10 s and the last 10 s in order to remove any unstable parts of the signal.

#### 2.3.2. Feature Definition and Extraction

To obtain features related to the characteristics of the tremors defined in the UPDRS guidelines, we defined 13 features from each of the signals (acceleration, angular velocity, displacement, and angle). The defined features can be categorized into temporal and spectral features. For the temporal features, the root mean square (RMS) was calculated first from the three-axis signal. In the frequency domain, three spectra from the three-axis signal were first calculated and then averaged at each frequency to obtain one spectrum reflecting the three-dimensional (3-D) tremor movement before extracting all of the features.

**Figure 3:**
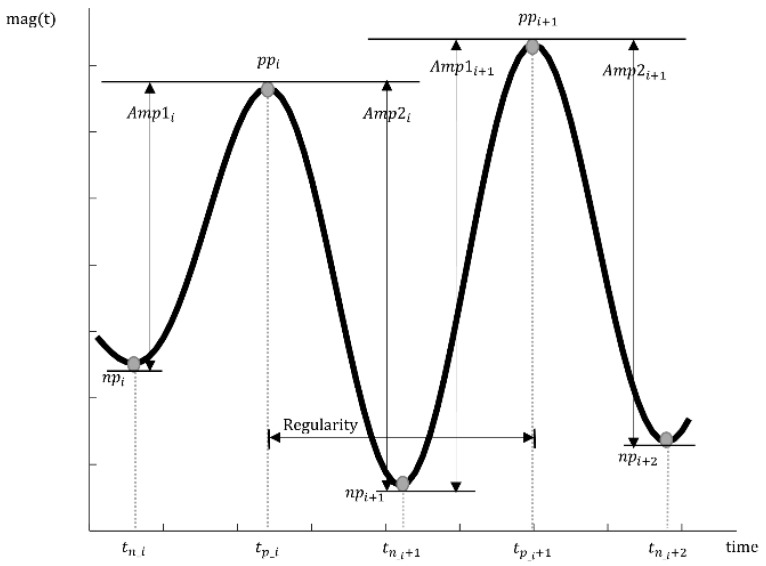
Graphically intuitive representation of temporal features. Two consecutive positive peaks (PP) and three consecutive negative peaks (NP) of the root mean square (RMS) signal of a tremor are shown together. The amplitude of each peak and the regularity are illustrated.

We first defined three features in the time domain. The mean amplitude of the tremor for a segmented period was calculated by averaging the peak-to-peak amplitude. In addition, the averaged regularity was computed to check the variability of the tremor rhythm. The standard deviation of the regularity for the segmented period was also added to the temporal features. These temporal features were calculated by the following equations and are illustrated graphically in Figure 3.

- Mean amplitude: average amplitude of a tremor for a segmented period
  (1) $$Amp1_{i}\;=\;|pp_{i}\;-\;np_{i}|$$
  (2) $$Amp2_{i}\;=\;|np_{i+1}\;-\;pp_{i}|$$
  where $pp_{i}$ = mag ($t_{p\_i})$, $np_{i}$ = mag ($t_{n\_i})$ (mag (*t*) is a function of the magnitude over time)
  (3) $$\mathrm{Mean}\;\mathrm{amplitude}\;=\;\frac{\sum_{i=1}^{n}Amp1_{i}+Amp2_{i}}{2n},\;i:\;theorderofpeak$$
- Average regularity: average time from the prior peak to the next peak
  (4) $$\mathrm{Average}\;\mathrm{regularity}\;=\;\frac{\sum_{i=1}^{n-1}|\;t_{p\_i+1}\;-\;t_{p\_i}\;|}{n-1}$$

In the frequency domain, another 10 features were obtained. The peak frequency (PF), mean frequency (MF), peak power, and mean power were computed from an averaged spectrum. The peak frequency was defined as the frequency at the maximum power in an averaged spectrum. The mean frequency was defined as the value for assessing the center of the distribution of the power across frequencies. The peak power and mean power are the power values at the peak frequency and mean frequency, respectively.

- Mean frequency
  (5) $$\mathrm{Mean}\;\mathrm{frequency}\;=\;\frac{\sum_{i=1}^{n}\;f_{i}\;\cdot \;P_{i}\;}{\sum_{i=1}^{n}P_{i}}$$
  where *i*: a sample in a spectrum, *f_i_*: a frequency at a sample *i*, and *P_i_*: a power value at a sample *i*.

**Figure 4:**
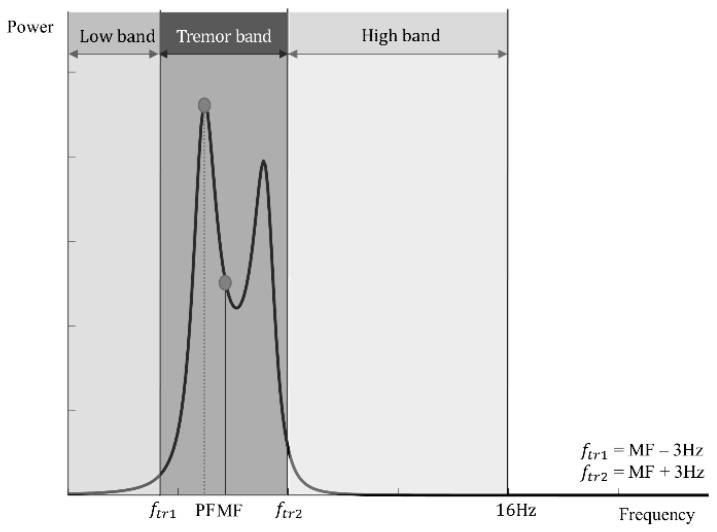
Graphically intuitive representation of the three frequency bands and spectral features in an averaged spectrum. The peak frequency (PF) and mean frequency (MF) are represented as an example. On the basis of the MF, the tremor frequency band is separated first; then, the remaining low- and high-frequency bands are determined.

The other six features were derived from three frequency bands in an averaged spectrum, as listed in Table 3 [19]. To calculate these features, we first defined the variable frequency bands on the basis of the mean frequency. The tremor frequency band was defined on the basis of the 3 Hz preceding ($f_{tr1}$ and the 3 Hz ($f_{tr2}$ following the mean frequency. Then, the low-frequency band was set to range from 0 Hz to $f_{tr1}$ and the high-frequency band from $f_{tr2}$ to 16 Hz, our last interesting frequency for the analysis of tremor characteristics. These three frequency bands are illustrated in Figure 4. We did not fix each of the three frequency bands, considering that each patient has a different mean frequency. In each of the three frequency bands, we calculated the power values in the low-frequency band (*P_Low_*), the tremor-frequency band *(P_Tr_*), and the high-frequency band (*P_High_*). The relative power values (*P_rl_*) in these frequency bands were also computed. Relative power values signify the ratios of each frequency band to the total power value from 0 Hz to 16 Hz. In addition to the 13 features described thus far, we added the logarithms of the mean amplitude (log(mean amplitude)), the peak power value (log(peak power)), the mean power value (log(mean power)), and power values in the three frequency bands: (log(*P_low_*), log(*P_tr_*), and log(*P_High_*)). As a result, 19 features were calculated from each set of four signals.

With these features of the four signals described thus far, we implemented the pairwise selection strategy to reduce the dimension of the features [33,34,35]. All possible correlation coefficients of each feature were first calculated; then, the features were arranged in order of increasing correlation coefficient from the smallest correlation, which means that the correlation coefficient between the first two features is the minimum among all possible correlation coefficients. The selected features are determined in the most unrelated order. By adding the selected features in order, the optimized feature configuration was determined when the highest accuracy was achieved. By using a principal component analysis (PCA), newly projected data are acquired by transforming all of the original variables with the principal components, i.e., a linear combination of the original variables [36]. The information obtained by the PCA is not redundant. The feature set obtained by two feature selection methods was applied to the machine-learning classifiers as input features; then, the optimal feature configuration for the classifiers was determined at the highest accuracy.

#### 2.3.3. Classification and Performance

An automatic scoring system for the tremor severity based on the kinematic features described in Section 2.3.2 was developed using several machine-learning algorithms: a decision tree, an SVM, a discriminant analysis, a random forest, and a *k*-nearest-neighbor (*k*NN) algorithm. When we employed an SVM, three kernels (linear, polynomial, and radial basis function (RBF)) were used. When we employed the *k*NN classification method, odd numbers between 1 and 11 were considered for the *k* values. The reason why odd numbers were used is to avoid the ambiguity of selection when a *k* value is even. For validation, a leave-one-out cross-validation method was adopted to avoid bias in the classification performance (five UPDRS classes were trained using different machine-learning methods in this study. With limited samples, leave-one-out is the most suitable method to train all classes without bias.). One data point among the dataset was used, in turn, for a prediction of the UPDRS, and the remaining data points were used to train the classifiers. The trained classifiers returned the estimated UPDRS classes of the tested points. We defined an absolute UPDRS classification error to evaluate the performance of the automatic scoring systems. The classification error is as follows [26]:

(6) $$e≜\left|\;\hat{u}-u\;\right|$$

where u is a real UPDRS score assigned by two neurologists, and $\hat{u}$ is a predicted class of tremors as determined by a trained classifier. We calculated the cumulative distribution functions (CDFs) of the error e to assess the classification accuracy and the probability for estimation within the error value. The root mean square error (RMSE) was also computed to evaluate the performance of the classifiers. All offline analyses were carried out using MATLAB R2016b (MATLAB, Mathworks, Natick, MA, USA).

## 3. Results

**Figure 5:**
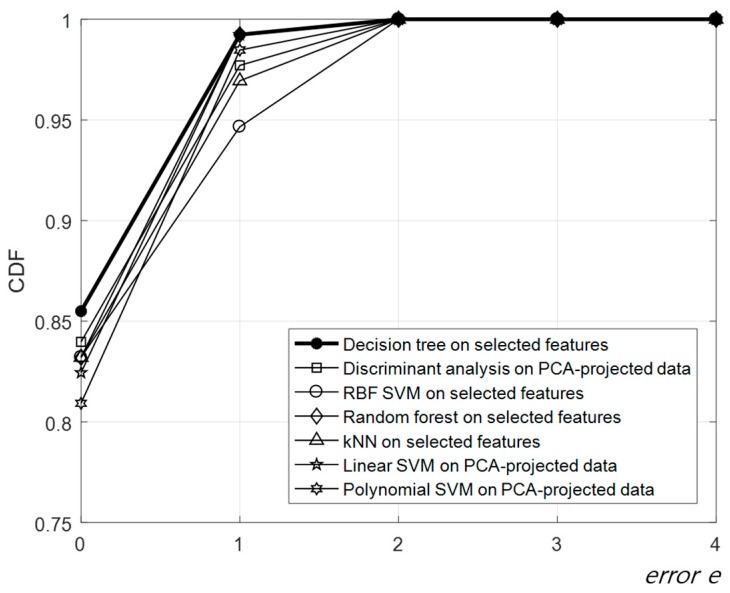
Cumulative distribution functions (CDFs) of the classification error *e* of each optimized classifier. The thick black line with filled circles is the best result obtained by the decision tree for the PCA-projected data.

The CDFs of the classification error *e* of each optimized classifier for the selected features are shown in Figure 5. The ability of the classifiers to predict the UPDRS ratings with each *e* value is shown, where the CDF values on the *Y* axis indicate the probability of obtaining a prediction with *e* ≤the corresponding *X* value. The highest accuracy of the automatic scoring of tremor severity corresponding to the CDF values at *e* = 0 was 85.5% and was achieved with the decision tree for the selected features by the pairwise correlation method. The lowest accuracy was 80.92%, obtained by the polynomial SVM for the PCA-projected data. The probability of an estimate with *e* ≤ 1 ranged from 94.66% to 99.24% depending on the classifiers. The decision tree returning the highest accuracy showed the highest probability of 99.24%, which means that the tremor severity can be estimated within a difference of one compared to the clinician’s decisions with a probability of 99.24% using our proposed method. The random forest and linear SVM also showed the same probability of 99.24%, although their accuracies were lower than that of the decision tree. The areas under the curve (AuCs) of the CDFs were also calculated. Here, we defined the normalized area under the curve (NAuC) of a CDF as the AuC divided by the total area. The performance, including the RMSE of each optimized classifier, is summarized in Table 4. This performance in Table 4 was determined for the optimized feature configuration producing the highest accuracy. The features selected by the pairwise correlation method and PCA were added to each classifier in order; then, the optimized feature configuration was determined at the highest accuracy of each classifier, as mentioned above. The dimension of the optimized feature configuration for each classifier is presented in Table 4. The dimension of the features for the PCA was determined to be two for the highest accuracy of all corresponding classifiers, and the cumulative variance for PC1–PC2 was 66.9%. As for the NAuC, the performance of all automatic scoring systems was similar with a range 0.971–0.980 (STD = ± 0.0046). In terms of the RMSE, the minimum error, 0.034, was achieved with the decision tree, and the largest error, 0.040, was obtained with the polynomial SVM. The deviation of the RMSE was also very small (STD = 0.0023), such as that for the NAuC. The performance results in Table 4 were arranged in order of accuracy. Note that the decision tree showed the highest accuracy and NAuC, and the lowest RMSE, for the five selected features. In conclusion, the decision tree was selected as the best classifier for an automatic scoring system of tremor severity with the highest accuracy of 85.55%, a NAuC of 0.980, and the smallest error of 0.034 among all explored classifiers.

The performance of the automatic scoring system was investigated further with a confusion matrix from the automatic predictions of the decision tree. The recalls and precisions are presented in Table 5 along with the 95% confidence intervals. The classifier determined UPDRS 0, 1, 2, 3, and 4 with recalls of 0.949 (±0.038), 0.818 (±0.066), 0.682 (±0.080), 0.667 (±0.081), and 0.000, respectively. The precisions for the UPDRS from 0 to 3 were 0.938 (±0.041), 0.720 (±0.077), 0.882 (±0.055), and 0.444 (±0.085), respectively. The precision for UPDRS 4 was undefined because all instances of UPDRS 4 were predicted as UPDRS 3. It seems that our proposed method tends to predict the UPDRS with a bias toward UPDRS 0–2, which are relatively dominant classes in terms of the amount of tremor data. These results may be connected with the number of patients corresponding to each UPDRS class. Considering the distribution of PD patients with tremor symptoms who visit the hospital, there are many more patients with UPDRS 1 and 2 than UPDRS 3 and 4. This distribution may affect the training ability of machine-learning classifiers. Obviously, a larger amount of tremor data with the distribution of each UPDRS would allow more relevant results to be achieved (from a statistical point of view) [24]. This will be explained in detail in Section 4.2.

## 4. Discussion

### 4.1. Main Findings

This paper proposed an alternative approach for the development of an automatic scoring system for tremors in PD using machine-learning algorithms. Compared to the existing methods, our approach can provide an estimated UPDRS, similar to the UPDRS already used in clinical practice with a high accuracy and the smallest margin of error yet. Plenty of researchers have studied tremor analysis to provide informative features with high and significant correlations to the UPDRS [10,11,18], with linear and nonlinear characteristics of tremors [9] to detect Parkinsonian tremors [11], and to distinguish Parkinsonian tremors from healthy people or other tremor symptoms such as essential tremors [8,9,14,16,19]. However, these studies may have difficulties in directly applying their methods to clinical practice for clinicians who evaluate tremors, since their results do not provide the same type of UPDRS. Recently, several studies about automatic UPDRS scoring have been conducted, but these studies were for gait-related tasks and bradykinesia [22,23,24,25,26,27]. As mentioned in Section 1.2, all other studies of automatic scoring for PD tremors exploited a regression model [30,31,32]. Therefore, this is the first study using a machine-learning approach to automatically predict the UPDRS of the Parkinsonian tremor severity, to the best of our knowledge. Compared to [30], the study reported an RMSE of 0.32 to estimate UPDRS 0–4 for resting tremors in 87 tremor trials using a linear regression model. Our proposed method using machine-learning algorithms showed an RMSE of 0.034 for the automatic scoring of resting tremors using 131 tremor recordings. In [31] and[32], the specific qualifiers for automatic scoring, such as the accuracy or RMSE, were not shown. Therefore, the machine-learning algorithms utilized in this study present the most accurate and reliable findings out of all of the related studies referenced based on our results and are the most logical candidates for use in a clinical or home context.

### 4.2. Limitations

This proposed system focused on the automatic scoring of resting tremors for PD patients. This system should be expanded to include not only other tremor tasks such as postural tremors and kinetic tremors but also motor fluctuations of other extremities of patients as alternatives to classic methods in clinical practice for the quantitative and objective diagnosis of the movement disorders of PD patients. We plan to carry out related work with a more relevant dataset to develop a more perfect automatic scoring system of tremor severity with continuous feedback from neurologists working in the Department of Neurology at SNUH in the future.

There are other limitations related to the underestimation of our proposed method. The prediction of each UPDRS tended to be biased toward UPDRS 0–2. Lower recalls and precisions resulted in a higher UPDRS, as presented in Table 5. Particularly, the recall of UPDRS 4 was 0, and the precision of UPDRS 4 was undefined, since all instances of UPDRS 4 were not correctly predicted. The nonuniform distribution of UPDRS 0–4 may affect the ability of classifier models to train and predict the UPDRS classes, since a larger number of samples would be connected to obtain more relevant results, as mentioned above. The employed classifiers had a strong tendency to ignore the UPDRS classes with a small amount of tremor data, particularly UPDRS 4, and predict them close to the dominant class. Moreover, the agreement between the UPDRS scores provided by two neurologists was particularly low at high UPDRS 3 and 4, as shown Figure 2. This also may lead to misclassification of the machine-learning classifiers. These elements are considered as our limitations resulting in underestimation by the classifiers in general.

However, it is an inevitable occurrence, due to the population of patients with tremor symptoms in a typical hospital [24]. There tend to be fewer patients with UPDRS 3 and 4 than UPDRS 1 or 2 in clinical practice because most patients with severe tremors are treated through medication or surgery. As a result, a reduced distribution for UPDRS 3 and 4 tremors is an inevitable and necessary consequence. Note that a lower or even zero distribution for UPDRS 3 and 4 has been reported in other similar studies [24,25,30].

Finally, the wearable device was used for the measurement of tremor symptoms in this study, but the incorporation of the proposed method on a wearable device may be considered in the future study. In this case, the required computational complexity on the wearable device should be taken into account for the online analysis of tremor severity on the wearable device.

## 5. Conclusions

This paper presents an alternative approach for the development of an automatic scoring system for the PD tremor using machine-learning approaches. We explored the use of a decision tree, an SVM with three kernels, a discriminant analysis, a random forest, and a *k*NN algorithm for the selected features by a pairwise correlation strategy. These machine-learning algorithms were validated using a leave-one-out method and evaluated with various statistical methods. The main finding is a highly accurate automatic scoring system for Parkinsonian tremor severity using machine-learning methods. An RMSE of 0.034 was obtained for the measurement of five classes of the UPDRS compared to the traditional UPDRS measured by neurologists. This error is less than those of other methods that have been proposed, and the performance of our method has an accuracy of 85.5%, which is the best accuracy to the best of our knowledge for the automatic scoring of Parkinsonian tremors.

The principal advantage of our approach is that it provides a predicted UPDRS with a high accuracy using a wearable device, which is most similar to neurologist-evaluated tremor scores in routine clinical practice using classical rating methods and can effectively be used in a home environment. Considering that existing studies have not yet been applied to real clinical practice in spite of the promising results, the application of the proposed method as a decision support tool is expected to naturally bridge new technology and the current subjective clinical rating scales in order to phase-out the present subjective clinical rating scales. Ultimately, the proposed approach could be highly useful for the effective diagnosis and management of PD.

## Figures and Tables

**Table 1:** Unified Parkinson’s Disease Rating Scale (UPDRS) for a tremor at rest (head, upper, and lower extremities).

Score	Guide
0	Absent
1	Slight and infrequently present
2	Mild in amplitude and persistent, or moderate in amplitude but only intermittently present
3	Moderate in amplitude and present most of the time
4	Marked in amplitude and present most of the time

**Table 2:** Dimensions and weight of the designed wearable device.

	Dimensions	Weight
Finger part	16 mm × 19.9 mm × 10 mm	2.6 g
Wrist part	41 mm × 48 mm × 17.8 mm	31.6 g

**Table 3:** Features derived for the three frequency bands.

Features	Definition
Power in low-frequency band ($P_{Low})$	$P_{Low}$ = $\sum_{i=1}^{i_{-tr1}}P_{i}$
Power in tremor-frequency band ($P_{Tr})$	$P_{Tr}$ = $\sum_{i=i_{-tr1}}^{i_{-tr2}}P_{i}$
Power in high-frequency band ($P_{High})$	$P_{High}$ = $\sum_{i=i_{-tr2}}^{i_{-16}}P_{i}$
Relative power in low-frequency band ($P_{rl\_Low})$	$P_{rl_{Low}}$ = $\frac{P_{Low}}{\sum_{i=1}^{n}P_{i}}$
Relative power in tremor-frequency band ($P_{rl\_Tr}$)	$P_{rl_{Tr}}$ = $\frac{P_{Tr}}{\sum_{i=1}^{n}P_{i}}$
Relative power in high-frequency band ($P_{rl\_High})$	$P_{rl_{High}}$ = $\frac{P_{High}}{\sum_{i=1}^{n}P_{i}}$

**Table 4:** Performance of each optimized classifier *.

Classifiers	Feature Selection Method	Acc. (%)	NAuC	RMSE
**Decision Tree**	MF, $P_{High}$, Mean power, $P_{rl\_Low}$, PF	**85.55** **(±6.03** ^†^**)**	**0.980**	**0.034**
Discriminant Analysis	PC1–PC2	83.97 (±6.28)	0.977	0.037
RBF SVM	MF, $P_{High}$	83.21 (±6.40)	0.977	0.037
Random Forest	MF, $P_{High}$, Mean power	83.21 (±6.40)	0.971	0.039
*k*NN (No. of neighbors = 3)	MF, $P_{High}$	83.21 (±6.40)	0.966	0.041
Linear SVM	PC1–PC2	82.44 (±6.52)	0.972	0.039
Polynomial SVM	PC1–PC2	80.92 (±6.73)	0.972	0.040

* The contents of this table are arranged in order of accuracy. ^†^ The 95% confidence intervals are provided for accuracy in parentheses.

**Table 5:** Confusion matrix of the UPDRS predicted by the proposed method.

		Predicted					
		0	1	2	3	4	Recall
**True**	0	**75**	4	0	0	0	0.949 (±0.038 *)
	1	4	**18**	0	0	0	0.818 (±0.066)
	2	1	3	**15**	3	0	0.682 (±0.080)
	3	0	0	2	**4**	**0**	0.667 (±0.081)
	4	0	0	0	2	**0**	0 (±0.000)
	Precision	0.938 (±0.041)	0.720 (±0.077)	0.882 (±0.055)	0.444 (±0.085)	undefined	

* The 95% confidence intervals are provided for all recalls and precisions in parentheses.
