# Correction: Automatic Classification of Tremor Severity in Parkinson’s Disease Using a Wearable Device. *Sensors* 2017, *17*, 2067

**DOI:** 10.3390/s18010033

**Published:** 2017-12-24

**Authors:** Hyoseon Jeon, Woongwoo Lee, Hyeyoung Park, Hong Ji Lee, Sang Kyong Kim, Han Byul Kim, Beomseok Jeon, Kwang Suk Park

**Affiliations:** 1 The Interdisciplinary Program for Bioengineering, Seoul National University, Seoul 03080, Korea; nulpurunhs@bmsil.snu.ac.kr (H.J.); hongjidan@bmsil.snu.ac.kr (H.J.L.); skkim@bmsil.snu.ac.kr (S.K.K.); hahanbyul@bmsil.snu.ac.kr (H.B.K.); 2 Department of Neurology and Movement Disorder Center, Seoul National University Hospital, Seoul 03080, Korea; w2pooh@daum.net (W.L.); 0907bluelove@naver.com (H.P.); brain@snu.ac.kr (B.J.); 3 Department of Biomedical Engineering, Seoul National University College of Medicine, Seoul 03080, Korea

The authors would like to make the following corrections to their paper [1]:

1. In page 8, “In terms of the RMSE, the minimum error, 0.034, was achieved with the decision tree, and the largest error, 0.040, was obtained with the polynomial SVM. The deviation of the RMSE was also very small (STD = 0.0023), such as that for the NAuC.” should be revised as “In terms of the RMSE, the minimum error, 0.410, was achieved with the decision tree, and the largest error, 0.573, was obtained with the RBF SVM. The deviation of the RMSE was also very small (STD = 0.054), such as that for the NAuC”.
2. In page 9, “the smallest error of 0.034 among all explored classifiers.” should be revised as “the smallest error of 0.410 among all explored classifiers.”
3. In page 9, RMSE values in Table 4. should be corrected as below:

4. In page 10, “the smallest margin of error yet” should be revised as “the smallest margin of error using the full range UPDRS data”.
5. In page 10, “UPDRS 0-4 for resting tremors” should be revised as “UPDRS 0-3 with a score interval of 0.25 for resting tremors”.
6. In page 10, “an RMSE of 0.034 for the automatic scoring of resting tremors using 131 tremor recordings.” should be revised as “an RMSE of 0.410 to predict full UPDRS range for resting tremor from 0 to 4 with a score interval of 1 using 131 tremor recordings. Neurologists practically use score interval of 1 in clinical practice”.
7. In page 11, “An RMSE of 0.034 was obtained for the measurement of five classes of the UPDRS compared to the traditional UPDRS measured by neurologists. This error less than those of other methods that have been proposed” should be revised as “An RMSE of 0.410 was obtained for the measurement of five classes of the UPDRS compared to the traditional UPDRS measured by neurologists. This error is the smallest for automatic scoring of full range UPDRS in resting tremor”.

The authors would like to apologize for any inconvenience caused by these changes.

## Figures and Tables

**Table 4:** Performance of each optimized classifier *.

Classifiers	Feature Selection Method	Acc. (%)	NAuC	RMSE
**Decision Tree**	MF, *P_High_*, Mean power, *P_rl_Low_*, PF	**85.55** **(±6.03** ^†^**)**	**0.980**	**0.410**
Discriminant Analysis	PC1–PC2	83.97 (±6.28)	0.977	0.479
RBF SVM	MF, *P_High_*	83.21 (±6.40)	0.977	0.573
Random Forest	MF, *P_High_*, Mean power	83.21 (±6.40)	0.971	0.437
kNN (no. of neighbors = 3)	MF, *P_High_*	83.21 (±6.40)	0.966	0.510
Linear SVM	PC1–PC2	82.44 (±6.52)	0.972	0.446
Polynomial SVM	PC1–PC2	80.92 (±6.73)	0.972	0.486

* The contents of this table are arranged in order of accuracy. ^†^ The 95% confidence intervals are provided for accuracy in parentheses.

## References

[1] Jeon H., Lee W., Park H., Lee H., Kim S., Kim H., Jeon B., Park K. (2017). Automatic Classification of Tremor Severity in Parkinson’s Disease Using a Wearable Device. Sensors.

